# Analysis of the correlation between serum Klotho and FeNO: a cross-sectional study from NHANES (2007–2012)

**DOI:** 10.1186/s12890-024-02864-7

**Published:** 2024-01-29

**Authors:** Haiyan Mao, Zhenye Xie, Shanshan Huang, Xingkai Shen, Shaofeng Jin, Tong Lin, Zhouxin Yang

**Affiliations:** 1 Department of Critical Care Medicine, Ningbo Medical Center Lihuili Hospital, 315100 Ningbo, China; 2 Zhejiang Provincial Key Lab of Geriatrics and Geriatrics Institute of Zhejiang Province, Department of Geriatrics, Zhejiang Hospital, 310030 Hangzhou, China

**Keywords:** Klotho, FeNO, Airway inflammation, Chronic respiratory diseases

## Abstract

**Background:**

Klotho is an anti-aging protein that has multiple functions and may play a key role in the pathogenesis and progression of chronic respiratory diseases such as chronic obstructive pulmonary disease (COPD). Fractional Exhaled Nitric Oxide (FeNO) is a non-invasive and novel biomarker that has the advantages of being simple, fast and reproducible. It can effectively assess the degree of airway inflammation in diseases such as asthma and COPD. Despite these insights, the relationship between serum Klotho levels and FeNO has not been explored yet.

**Methods:**

Leveraging data from the National Health and Nutrition Examination Survey (NHANES) spanning 2007 to 2012, we investigated the correlation between FeNO and serum Klotho levels. This association was scrutinized both as continuous variables and within quartile distributions, utilizing the Kruskal-Wallis H test. The correlation between the two variables was assessed through Spearman rank analysis. Employing survey weight-adjusted linear regression models, we gauged the strength of these associations.

**Results:**

This study included 6,527 participants with a median FeNO level of 14.5 parts per billion (ppb). We found that FeNO levels varied significantly across different quartiles of Klotho protein (H = 7.985, *P* = 0.046). We also found a significant positive correlation between serum Klotho levels and FeNO levels in the whole population (Spearman’s rho = 0.029, *P* = 0.019). This correlation remained significant after adjusting for covariates such as age, gender, lung function, smoking status, alcohol use, BMI, cardiovascular disease (including hypertension, heart failure, coronary heart disease, and myocardial infarction), diabetes, inflammatory markers, serum vitamin D level and BUN (*P* < 0.05 for all). Furthermore, this correlation was stronger at the high (K3) and super high (K4) levels of Klotho than at the low (K1) and medium (K2) levels (β = 1.979 ppb and β = 1.993 ppb for K3 and K4 vs. K1, respectively; 95% CI: 0.497 ~ 2.953 and 95% CI: 0.129 ~ 2.827, respectively; *P* = 0.007 and *P* = 0.032, respectively). The β coefficient for serum Klotho was 0.002 ppb/pg/ml.

**Conclusions:**

Our study illuminates a positive correlation between serum Klotho levels and FeNO. Further study is needed to verify the causality of this association and elucidate the underlying mechanisms.

**Supplementary Information:**

The online version contains supplementary material available at 10.1186/s12890-024-02864-7.

## Introduction

Chronic respiratory diseases such as COPD and asthma have a huge economic impact on the world, involving medical expenses, productivity losses, and social welfare payments. COPD is one of the top three killers worldwide, causing 3.23 million deaths in 2019 as per WHO. The annual direct medical expenses of COPD are more than $38 billion, while the indirect expenses (such as missing work, retiring early, being disabled, etc.) amount to $200 billion. The annual direct medical expenses of asthma are more than $19 billion, while the indirect expenses (such as missing work, retiring early, being disabled, etc.) amount to $100 billion [1–4]. These chronic respiratory diseases are manageable but incurable, and they require different treatment approaches depending on the disease progression.

The Klotho gene is an anti-aging gene discovered by Japanese scholar Kuro-o in 1997 while studying spontaneous hypertension [5]. The Klotho gene family consists of the Klotho gene, also known as the α-Klotho gene, the β-Klotho gene, and the Klotholactase-phlorizin hydrolase (Klph) gene. The Klotho gene encodes a 135 kDa type I transmembrane protein called Klotho protein, also known as α-Klotho protein, which contains 1,012 amino acids [6]. Soluble α-klotho (hereinafter referred to as klotho), which sheds its amino-terminal extracellular domain, circulates in the blood, urine, and cerebrospinal fluid [7, 8]. It is a hormone with various health-related functions, such as enhancing insulin sensitivity and glucose uptake, regulating endothelial nitric oxide synthesis, reducing oxidative stress and inflammation [9–11]. It plays an important role in the pathogenesis and progression of respiratory diseases such as COPD, asthma, pulmonary fibrosis, lung cancer and more [12–17]. Therefore, Klotho protein may offer a novel therapeutic strategy for the prevention and treatment of respiratory diseases.

Fractional exhaled nitric oxide (FeNO) is a novel method for detecting airway inflammation, which uses the average concentration of eNO to identify the inflammation type. This test is simple, non-invasive, standardized, and can be performed repeatedly at different phases of the disease [18–21]. When the airway is inflamed, the airway epithelial cells increase the expression of inducible nitric oxide synthase (NOS2), which produces more nitric oxide (NO). These NO molecules are exhaled with the breath, so the NO in the exhaled air mainly originates from the airway epithelium [22–29]. Different lung diseases (such as asthma, atopy, chronic obstructive pulmonary disease, fibrosing alveolitis, radiation pneumonitis, viral infections, etc.) affect the levels of FENO in patients. Several epidemiological studies have shown that the FENO levels of these patients are usually elevated [20, 21].

A large number of studies have confirmed that klotho play an important role in respiratory diseases, while FeNO can reflect the level of airway inflammation, but there is no report on the relationship between klotho protein level and FeNO at present. This study aims to explore the correlation between them.

## Materials and methods

### Data source

This is a cross-sectional analysis that used data from the National Health and Nutrition Examination Survey (NHANES) (https://www.cdc.gov/nchs/nhanes). NHANES is a nationwide survey conducted by the Centers for Disease Control and Prevention (NCHS) that combines interviews and physical examinations. The NHANES uses a complex, multistage design to gather and analyze data that reflects the US’s noninstitutionalized national population. The study execution involved two pivotal components: comprehensive interviews conducted within participants’ homes and meticulous health examinations conducted at mobile examination centers. Both segments of the study were meticulously executed by proficient and certified researchers.

### Ethics considerations

The NCHS Research Ethics Review Board gave their approval for the NHANES program, and all the survey participants agreed to sign a consent form. The NCHS allows researchers to use the data that they make available for research purposes. The data from NHANES are also anonymized by the NCHS before they are released to the public, and the data remain anonymous during the analysis. Therefore, no additional ethical approval or informed consent was needed for the secondary data analysis that we conducted in this study. The details of the NCHS Research Ethics Review Board Approval can be found on the NHANES website (https://www.cdc.gov/nchs/nhanes/irba98.htm).

### Study design and participants

**Fig. 1:**
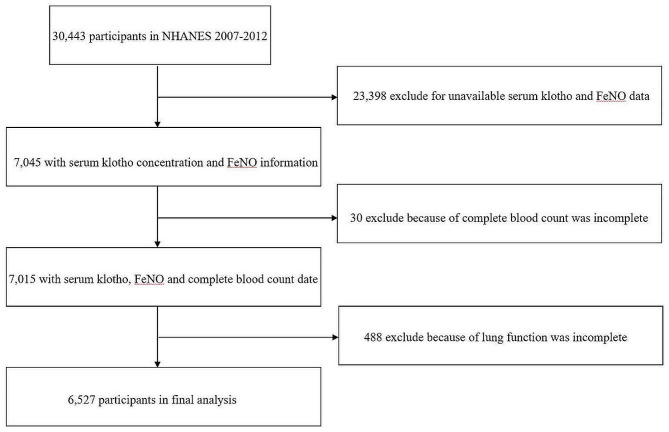
Screening conditions and process for the study population

In this study, we analyzed the data from the three NHANES cycles between 2007 and 2012, because only these three cycles had both serum klotho and FeNO data. A total of 30,443 people in the United States participated in the survey between 2007 and 2012. However, among this cohort, 23,398 participants were excluded from the overall sample due to non-availability of Klotho or FeNO measurements. Further refinement was performed by excluding individuals with incomplete data on key variables such as complete blood count parameters (including white blood cell count, lymphocyte count, eosinophil count, segmented neutrophil count) and lung function metrics (FEV1, FVC, PEF, FEF25%-75%, FEV1/FVC). This meticulous screening left us with a final eligible sample size of 6,527 subjects, who then became the focal point of subsequent analyses. A visual representation detailing the study’s design and structure is thoughtfully presented in Fig. 1 for clarity.

### Measurement of serum Klotho and FeNO

Serum specimens from participants aged 40–79 years old collected, flash-frozen, NHANES reported that serum samples from participants aged 40–79 years were collected, flash-frozen, and stored at -80 °C at the Centers for Disease Control and Prevention. The Northwest Lipid Metabolism and Diabetes Research Laboratories at Washington University received these samples in 2019–2020. They used a commercially available ELISA kit from IBL International to measure individual α-Klotho concentrations [30]. The laboratories followed the manufacturer’s protocol, calculating mean values from duplicate sample analyses for α-Klotho concentrations and using standard samples to assess assay linearity. The minimum detection limit was determined to be 4.33 pg/ml, and the intra-assay and inter-assay coefficients of variation were within acceptable ranges. Results were recorded in the laboratory’s Oracle Management System and thoroughly reviewed.

The measurement of FENO was sponsored by the National Heart, Lung, and Blood Institute of the National Institute of Health. Individuals between the ages of 6 and 79 years were eligible for FENO testing. However, participants with current chest pain, physical issues related to forceful expiration, or those who were receiving supplemental oxygen were excluded from the study for medical reasons. FENO was measured using a handheld device (NIOXMINO, Aerocrine, Stockholm, Sweden) with an electrochemical sensor which was approved by the FDA in 2008. According to ATS/ERS guidelines [31], a valid measurement required two reproducible measurements at an expiratory flow rate of 50 ml/s, with no recent use of oral or inhaled steroids, no oxygen requirement, no difficulty in taking deep breaths, and no strenuous exercise in the hour preceding the measurement. Up to four attempts were made to measure FeNO, and the mean of two reproducible measurements (within 2 ppb if levels were < 30 ppb or within 10% if levels were > 30 ppb) was considered the final result. The NIOX MINO device has a lower detection limit of 5 ppb and an upper limit of 300 ppb. If two measurements were below the device’s detection limit, a value of 3.5 ppb (lower limit of detection divided by the square root of two) was used as the mean.

### Variables of interest

To measure the hematologic parameters, the NHANES Complete Blood Count (CBC) Profile was be used, which is based on the Beckman Coulter method of counting and sizing the blood cells, along with an automated device for sample dilution and mixing, and a single beam photometer for hemoglobin measurement. The white blood cell (WBC) count differential was performed using VCS technology. We calculated the neutrophil percentage/lymphocyte ratio (NLR) for each participant by dividing the absolute neutrophil counts by the absolute lymphocyte counts [32]. The DXC800 was used to measure the standard biochemical profile, including albumin, Alanine Aminotransferase (ALT), Aspartate Aminotransferase (AST), blood urea nitrogen (BUN), creatinine, and cholesterol. The DXC800 is a modular chemistry analyzer that uses various methods to quantify the concentration of different analytes in serum or plasma. Albumin, ALT, AST and creatinine are measured by enzymatic methods, BUN is measured by an enzymatic conductivity rate method, and cholesterol is measured by a cholesterol esterase/cholesterol oxidase method. The LX20 system uses indirect (or diluted) ISE methodology to measure calcium and phosphorus concentration in serum. The LX20 with LD reagent (using lactate as substrate) utilizes an enzymatic rate method to measure LD activity in biological fluids. Vitamin D can affect the expression level of klotho [33], so this study also included vitamin D as a covariate. A validated and standardized liquid chromatography-tandem mass spectrometry (LC-MS/MS) method was employed to measure both 25-hydroxyvitamin D3 [25(OH)D3] and 25-hydroxyvitamin D2 [25(OH)D2]. This method was utilized for all eligible participants in NHANES 2007–2014. The total serum vitamin D, which includes the sum of 25(OH)D3 and 25(OH)D2, was determined using this approach [34, 35]. The spirometry measurement procedures aligned with the recommendations of the ATS/ERS [36]. Baseline spirometry data from the initial test were utilized. This study incorporated a number of parameters to assess lung function: forced expiratory volume in the first second (FEV1), forced vital capacity (FVC), peak expiratory flow (PEF), forced expiratory flow at 25-75% of FVC (FEF25%-75%) and the ratio of FEV1 to FVC (FEV1/FVC).

### Demographic and medical information

Trained examiners collected demographic information during in-home interviews. The body mass index (BMI) was calculated as weight (kg) divided by height (m) squared (kg/m2). A history of smoking was defined as having smoked 100 or more cigarettes during one’s lifetime. Alcohol use history was confirmed by inquiring whether participants had consumed 12 or more alcoholic drinks in a year. Histories of medical conditions were verified by asking participants if they had been diagnosed with diabetes, hypertension, heart failure, coronary heart disease, or myocardial infarction by a doctor.

### Statistical analyses

The data underwent analysis utilizing SPSS 21.0 and R 4.0.0. Initial normality assessment through the Kolmogorov-Smirnov test indicated non-normal distribution. For non-normally distributed measurement data, median and interquartile range were employed for description, and group comparisons were made using the Kruskal-Wallis H test. Proportions were used for ordinal data description and comparisons, with the chi-square test used for categorical data. Klotho, a continuous variable, was divided into quartiles for analysis. Spearman rank correlation analysis was conducted to examine variable correlations. Partial correlation analysis was used to assess the relationship between klotho levels and feno, controlling for other confounding variables. Survey weight-adjusted linear regression analysis yielded beta coefficients to estimate correlation strength, and the R forestplot illustrated in a forest plot. Four models were provided: Model 1 (unadjusted Klotho), Model 2 (Klotho adjusted for relevant covariates), Model 3 (unadjusted Klotho quartiles), and Model 4 (Klotho quartiles adjusted for relevant covariates).

## Results

The baseline characteristics of this study population are shown in Table 1. This study engaged a participant cohort of 6,527 individuals, comprising 3,311 (50.7%) males and 3,216 (49.3%) females, with a mean age of 56 years. The median FeNO level measured 14.5 parts per billion (ppb). The median FeNO of the smoking group was 8.25 ppb, lower than that of the non-smoking group (8.25 vs. 16.50 ppb, *P* < 0.001). Klotho levels in the smoking group were not significantly different from those in the non-smoking group (789.20 vs. 791.90 pg/ml, *P* = 0.939). Notably, significant variations in FeNO levels were evident across quartiles based on Klotho protein levels (H = 7.985, *P* = 0.046). The first quartile (Q1) demonstrated considerably lower FeNO levels compared to the remaining three quartiles (13.5 vs. 14.5 ppb). Distinct differences in lung function were also noted across quartiles determined by Klotho protein levels (*P* < 0.05). Specifically, the second quartile (Q2) exhibited the highest FEV1, FVC, and PEF levels, while the fourth quartile (Q4) displayed significantly elevated FEF25%-75% and FEV1/FVC ratio compared to the other quartiles. There was a significant difference in serum klotho levels between groups (H = 12.935, *P* = 0.005). Among them, the Q3 group had the highest vitamin D level. Furthermore, Klotho protein levels displayed significant associations with WBC count, NLR, eosinophil (Eos) count, albumin, ALT, AST, BUN, lactate dehydrogenase (LDH), creatinine, history of alcohol consumption, history of heart failure, history of myocardial infarction and history of diabetes (*P* < 0.05 for all). However, Klotho levels exhibited no significant impact on participants’ weight (BMI), serum albumin, cholesterol, serum phosphorus, blood calcium, history of hypertension, history of coronary heart disease, or history of smoking.

**Table 1:** Study population characteristics by quartiles of Klotho protein

Variable	Total	Klotho Quartiles				H/χ^2^	P
		Q1(*n* = 1632)	Q2(*n* = 1633)	Q3(*n* = 1631)	Q4(*n* = 1631)		
FeNO(ppb)	14.5(9.5,21.5)	13.5(9,20.5)	14.5(9,21.5)	14.5(9.5,22)	14.5(9.5,21.5)	7.985	0.046
Age(years)	56(48,65)	58(49,67)	57(48,65)	56(48,65)	54(47,63)	43.969	0.000
Male,N(%)	3311(50.7)	862(52.8)	890(54.5)	822(50.4)	737(45.2)	32.260	0.000
BMI((kg/m2)	28.61(25.1,32.8)	28.67(25.2,32.83)	28.68(25.28,32.55)	28.6(25.1,32.7)	28.56(24.81,33.08)	0.599	0.897
WBC(*10^3/uL)	6.8(5.6,8.1)	6.9(5.7,8.4)	6.8(5.6,8.2)	6.7(5.6,8.1)	6.6(5.4,8)	25.362	0.000
NLR	1.95(1.48,2.58)	2(1.53,2.72)	2(1.5,2.54)	1.93(1.46,2.63)	1.88(1.42,2.48)	25.757	0.000
Eos(*10^3/uL)	0.2(0.1,0.3)	0.2(0.1,0.3)	0.2(0.1,0.3)	0.2(0.1,0.3)	0.2(0.1,0.2)	23.061	0.000
FEV1(mL)	2705(2186,3293)	2665.5(2138.25,3250)	2732(2231.5,3369)	2714(2181,3315)	2697(2190,3261)	14.109	0.003
FVC (mL)	3566(2895,4344)	3543(2847.25,4321)	3622(2943.5,4463)	3551(2918,4344)	3532(2868,4249)	14.644	0.002
PEF (mL/s)	7455(6072,9088)	7382.5(5956.75,8984)	7601(6168,9112.5)	7465(6049,9265)	7372(6063,9001)	8.127	0.043
FEF25%-75%(mL/s)	2331(1625,3155)	2199(1518.25,3026.25)	2351(1635,3224.5)	2332(1639,3153)	2415(1703,3213)	27.111	0.000
FEV1/FVC	77.02(71.76,81.46)	76.45(70.73,80.82)	76.86(71.9,81.18)	76.83(71.89,81.57)	77.95(72.59,82.02)	31.991	0.000
Albumin(g/L)	42(40,44)	42(40,44)	43(41,44)	42(41,44)	42(40,44)	26.179	0.000
ALT(U/L)	22(17,29)	21(17,28)	22(17,29)	22(17,29)	22(17,29)	10.956	0.012
AST(U/L)	24(20,29)	23(20,28)	24(21,29)	24(20,28)	24(20,29)	14.524	0.002
Cholesterol(mmol/L)	5.15(4.45,5.87)	5.15(4.37,5.97)	5.15(4.43,5.9)	5.17(4.47,5.84)	5.15(4.45,5.82)	0.980	0.806
BUN (mmol/L)	4.64(3.57,5.71)	4.64(3.57,6.07)	4.64(3.57,5.71)	4.64(3.57,5.71)	4.28(3.57,5.36)	42.918	0.000
Creatinine(mmol/L)	76.02(64.53,90.17)	79.56(65.42,93.7)	76.91(64.53,90.17)	76.02(64.53,88.4)	72.49(63.65,87.52)	59.019	0.000
LDH(U/L)	131(116,147)	129(115,147)	130(116,145)	131(116,146)	133(118,150)	18.874	0.000
Phosphorus(mmol/L)	1.2(1.07,1.32)	1.2(1.07,1.29)	1.2(1.1,1.32)	1.2(1.07,1.32)	1.2(1.1,1.29)	0.985	0.805
Calcium(mmol/L)	2.35(2.3,2.4)	2.35(2.3,2.4)	2.35(2.3,2.4)	2.35(2.3,2.4)	2.35(2.3,2.4)	3.750	0.290
Alcohol,N(%)	4578(73.9)	1227(78.6)	1177(75.3)	1126(73.5)	1048(68.4)	43.968	0.000
HBP, N (%)	2563(90.4)	685(90.8)	644(90.6)	622(90.7)	612(89.5)	0.935	0.817
Diabetes,N(%)						14.955	0.021
Yes, N (%)	990(15.2)	259(15.9)	230(14.1)	216(13.3)	285(17.5)		
No, N (%)	5366(82.3)	1331(81.6)	1356(83.2)	1378(84.5)	1301(79.8)		
Borderline, N (%)	166(2.5)	42(2.6)	44(2.7)	36(2.2)	44(2.7)		
Smoking, N (%)						3.781	0.706
Every day, N (%)	1138(34.7)	311(34.9)	295(34.1)	282(35.9)	250(34.1)		
Some day, N (%)	186(5.7)	57(6.4)	47(5.4)	36(4.6)	46(6.3)		
Not at all, N (%)	1951(59.6)	523(58.7)	524(60.5)	467(59.5)	437(59.6)		
Vitamin D(nmol/L)	64.40(46.60,81.50)	64.40(46.15,82.15)	64.5(48.40,82.10)	65.00(47.45,82.73)	62.20(44.80,78.85)	12.935	0.005
Heart failure, N (%)	179(2.8)	60(3.7)	50(3.1)	35(2.1)	34(2.1)	10.913	0.012
coronary heart disease, N (%)	260(4.0)	78(4.8)	69(4.2)	54(3.3)	59(3.6)	5.506	0.138
Myocardial infarction, N (%)	283(4.3)	88(5.4)	80(4.9)	57(3.5)	58(3.6)	10.767	0.013

### Analysis of the correlation between serum klotho levels and FeNO

A correlation analysis was conducted between Klotho levels and FeNO. Given the non-normal distribution of both variables, Spearman rank correlation analysis was employed. The results in Table 2 demonstrate a statistically significant Spearman rank correlation coefficient of 0.029 (*P* = 0.019) for the entire population, indicating a positive correlation. To ensure the observed correlation between serum Klotho levels and FeNO was independent of other covariates, a partial correlation analysis was performed, controlling for factors like WBC, NLR, Eos, and others. In all cases, the obtained *P* values were less than 0.05, reaffirming statistical significance.

**Table 2:** Correlation analysis between serum Klotho level and FeNO

Group	r_s/_r	P value
The entire population	0.029	0.019
Control WBC	0.025	0.045
Control NLR	0.027	0.027
Control EON	0.033	0.007
Control FEV1	0.026	0.035
Control FVC	0.027	0.030
Control PEF	0.026	0.035
Control FEF25%-75%	0.027	0.030
Control FEV1/FVC	0.028	0.025
Control Vitamin D	0.028	0.023

### Analysis of linear regression on serum klotho levels and FeNO

**Fig. 2:**
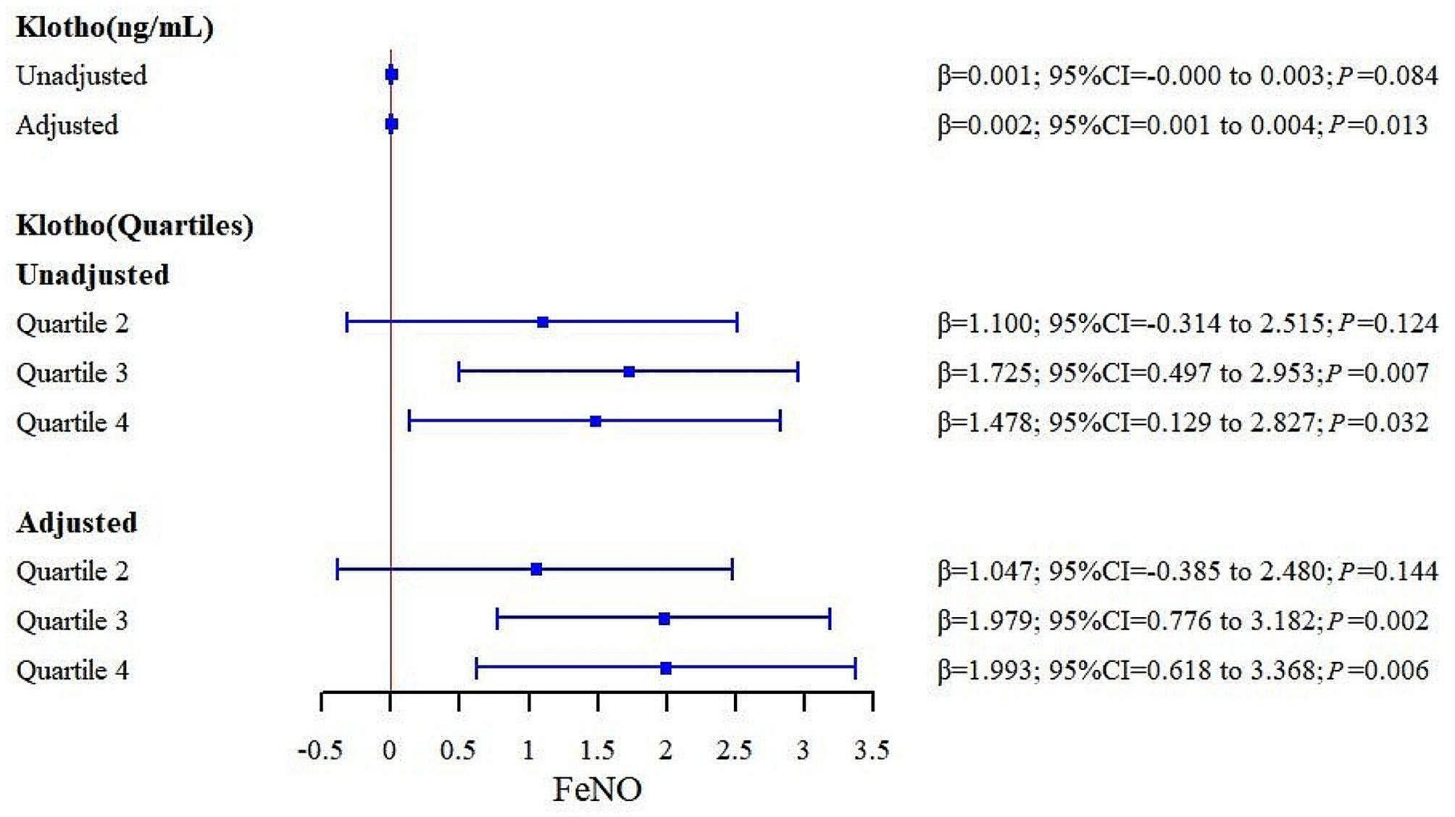
Forest plot of the β coefficients for the associations between serum Klotho level and FeNO. Serum Klotho was included in models as a continuous variable and as quartiles. Adjusted models included age, gender, BMI, WBC, NLR, eos, lung function, albumin, ALT, AST, BUN, creatinine, LDH, phosphorus, calcium, cholesterol, cardiovascular disease (including hypertension, heart failure, coronary heart disease and myocardial infarction), serum vitamin D level, diabetes, smoking status, and alcohol use. 95%CI, Confidence Interval

We performed a linear regression analysis to examine the relationship between serum Klotho levels, an anti-aging protein, and FeNO, a biomarker of airway inflammation. As shown in Table 3; Fig. 2, Klotho and FeNO were positively correlated, but this correlation was only significant at the high (K3) and super high (K4) levels of Klotho (95% CI: 0.497 ~ 2.953, *P* = 0.007 and 95% CI: 0.129 ~ 2.827, *P* = 0.032, respectively). After controlling for other confounding variables, the positive correlation between Klotho and FeNO became more significant (95% CI: 0.001 ~ 0.004, *P* = 0.013), and the β coefficient for serum Klotho was 0.002 ppb/pg/ml, indicating that a one-unit increase in serum Klotho was associated with a 0.002 ppb increase in FeNO. In addition, FeNO also increased with higher levels of Klotho. Compared to the lowest quartile (Q1) of Klotho, the β coefficients for the third (Q3) and fourth (Q4) quartiles were 1.979 ppb (95% CI: 0.776 ~ 3.182, *P* = 0.02) and 1.993 ppb (95% CI: 0.618 ~ 3.368, *P* = 0.006), respectively, indicating that the Q3 and Q4 groups had significantly higher FeNO levels than the Q1 group.

**Table 3:** Linear regression analyses for the associations between serum Klotho level and FeNO

Model		β	t	95.0%CI	P
Model 1	Klotho	0.001	1.766	-0.000,0.003	0.084
Model 3	K2	1.100	1.566	-0.314,2.515	0.124
	K3	1.725	2.828	0.497,2.953	0.007
	K4	1.478	2.205	0.129,2.827	0.032
Model 2	Klotho	0.002	2.672	0.001,0.004	0.013
Model4	K2	1.047	1.513	-0.385,2.480	0.144
	K3	1.979	3.404	0.776,3.182	0.002
	K4	1.993	2.998	0.618,3.368	0.006

Model 1: unadjusted Klotho, Model 2: Klotho adjusted for relevant covariates, Model 3: unadjusted Klotho quartiles, Model 4: Klotho quartiles adjusted for relevant covariates. 95%CI, Confidence Interval

## Discussion

In this study, we investigated the relationship between serum Klotho levels, an anti-aging protein, and FeNO, a biomarker of airway inflammation, in a large sample of American adults. We found a significant positive correlation between these two variables, indicating that higher Klotho levels are associated with higher FeNO levels. This correlation was especially strong among individuals with high Klotho levels. Moreover, this correlation persisted even after adjusting for a wide range of potential confounders, including age, gender, lung function, smoking status, alcohol use, BMI, cardiovascular disease (including hypertension, heart failure, coronary heart disease and myocardial infarction), diabetes, inflammatory markers, serum vitamin D level and BUN. This suggests that the association between Klotho and FeNO is not confounded by these factors and may reflect a causal relationship. To the best of our knowledge, this study stands as a pioneering effort in revealing and exploring this correlation within a diverse population. However, the precise mechanistic underpinnings of this connection remain an intriguing subject warranting further in-depth investigation.

Nitric oxide (NO) is an important signaling molecule produced in the human body that plays a crucial role in respiratory system diseases such as asthma, COPD, acute respiratory distress syndrome, pulmonary hypertension, pneumonia, and lung cancer. NO regulates the tone of blood vessels and bronchi, causing them to dilate, thereby improving gas exchange and reducing airflow resistance. It acts as a neurotransmitter, participating in the contraction and relaxation of airway smooth muscles, as well as activating airway sensory nerves. As an inflammatory mediator, it is involved in the occurrence and regulation of airway inflammation, particularly inflammation mediated by eosinophils. Additionally, NO serves as an antioxidant, protecting lung tissue from oxidative stress-induced damage [37, 38]. Epidemiological studies have shown that patients with respiratory system diseases such as asthma, atopic lung disease, COPD, fibrosing alveolitis, radiation pneumonitis, and viral infections have elevated levels of FeNO [20, 21]. FeNO is considered a useful, quantitative, non-invasive, and simple biomarker of airway inflammation, widely used in clinical settings [20, 21, 31]. However, FeNO measurements are influenced by various factors, including age, gender, smoking habits, dietary habits, rhinitis, infections, and medication use, which impose certain limitations [21, 39–44]. Hence, a comprehensive approach, combining multiple indicators, is necessary to aid clinicians in diagnosing and managing various respiratory conditions effectively.

Klotho is a multifunctional protein that possesses anti-inflammatory, antioxidant stress, and anti-apoptotic effects [9–11], participating in the physiological and pathological processes of various diseases in the human body. Several previous studies have demonstrated a close association between downregulation of Klotho expression and diseases such as COPD, cardiovascular diseases, diabetes, and kidney diseases [11, 45–49]. Our research findings also indicate significant differences in creatinine levels and the occurrence of diseases such as myocardial infarction, heart failure, and diabetes among individuals with different Klotho levels. Furthermore, Klotho can interact with vitamin D and fibroblast growth factor (FGF) family members involved in regulating calcium-phosphate metabolism balance, and deficiency or reduction of Klotho may lead to osteoporosis [33]. Additionally, our study results demonstrate significant differences in vitamin D levels between individuals with different Klotho levels (H = 12.935, *P* = 0.05), although the specific mechanisms require further investigation. It has been shown in previous studies that smokers’ FeNO is lower than that of healthy non-smokers [50], and this finding is consistent with our study. Additionally, Klotho levels are influenced by various factors [17], with smoking status being an important factor; however, the results of different studies are inconsistent [51–53]. Our research found no significant differences in Klotho concentrations between smokers and non-smokers, but this may be attributed to the self-reported method of obtaining smoking history, and the potential impact of smoking on Klotho expression levels cannot be excluded. Future studies should assess smoking intensity and duration more accurately to clarify the relationship between smoking and Klotho levels.

Klotho plays an important role in respiratory system diseases and may be a potential therapeutic target for airway diseases. COPD is considered a disease characterized by accelerated lung aging driven by chronic exogenous and endogenous oxidative stress [54]. Mice with Klotho gene deficiency exhibit features of COPD in the lungs, such as alveolar wall destruction, enlarged gas spaces, decreased elastic recoil, and prolonged expiratory time [55]. The expression of Klotho in the airway epithelium and serum is also reduced in COPD patients [52]. Airway inflammation and hyperresponsiveness are pathological features of asthma, and Klotho can inhibit the IGF-1 signaling pathway, which leads to reduced proliferation and contraction of airway smooth muscles, decreased airway hyperresponsiveness, and reduced airway resistance [49, 56]. Klotho can promote the synthesis and release of NO, thereby enhancing the vasodilation ability of blood vessels and airways, protecting against oxidative stress, and preserving endothelial function [57, 58]. Our study also demonstrates significant differences in FeNO levels between individuals with different Klotho levels. Furthermore, Klotho can inhibit signaling pathways such as Wnt/β-catenin and TGF-β1, reducing lung fibrosis in asthma patients and improving lung function [59]. Klotho can also inhibit the nuclear translocation of NF-κB and the expression of downstream genes, reducing the release of inflammatory mediators such as interleukin-6 and tumor necrosis factor-alpha, thereby alleviating airway inflammation in COPD and asthma patients [60]. Our results showed a negative correlation between Klotho levels and inflammatory factors (leukocytes, neutrophil/lymphocyte ratio), consistent with previous research findings [13, 61]. Klotho can improve airway mucociliary clearance by increasing airway surface liquid (ASL) volume and activating large-conductance calcium-activated potassium channels (BK) [62]. Additionally, Klotho may also play a significant role in the development of interstitial lung diseases, obstructive sleep apnea, and lung tumors. In a study on Klotho and interstitial lung abnormalities, serum Klotho levels were found to be not significantly different from the control group among patients with interstitial lung abnormalities, but they showed a positive correlation with lung function indicators such as FEV1, FVC, DLCO [14]. Our research also found a positive correlation between Klotho levels and FEV1/FVC, and significant differences in lung function among individuals with different Klotho levels. Serum Klotho levels are reduced in patients with obstructive sleep apnoea (OSA) due to chronic intermittent hypoxia, and Klotho may play a role in systemic inflammation in OSA [63]. Klotho can inhibit the IGF-1/insulin and Wnt/β-catenin signaling pathways, promoting autophagy and apoptosis of lung cancer cells, thus exerting an inhibitory effect on tumor growth [56].

### Limitations

Strengths of this study include its substantial sample size, representative of the community-dwelling population in the United States, and its meticulous adjustment for key confounders in the FeNO-Klotho relationship. However, the study also presents limitations. Its cross-sectional nature precludes the determination of temporal sequence. Given that serum Klotho levels were measured at a single time point, their reflection of long-term effects on FeNO might be limited. However, if such fluctuations are stochastic, they would tend to bias the effect size towards null, potentially underscoring a stronger association between Klotho and FeNO than observed. Secondly, Klotho is influenced by multiple factors, and although we have included as many confounding variables as possible, some potential unaccounted confounding variables such as medication history may still have an impact on the results. Additionally, as an observational study, causal inferences cannot be drawn. Therefore, in the future, more in-depth research is needed to elucidate the causal relationship and specific mechanisms of action between Klotho and FeNO.

## Conclusion

In conclusion, we found that serum Klotho levels and FeNO had a positive correlation. In addition, we also found that klotho levels were correlated with lung function (FEV1, FVC, PEF, FEF25%-75%, FEV1/FVC). These relationships require further in-depth research to elucidate the causal relationship and underlying mechanisms of this association.

### Electronic supplementary material

Below is the link to the electronic supplementary material.

Supplementary Material 1

## Data Availability

The data that support the findings of this study are available from the National Health and Nutrition Examination Survey (NHANES) (https://www.cdc.gov/nchs/nhanes).

## Abbreviations

COPD: Chronic obstructive pulmonary disease
FeNO: Fractional Exhaled Nitric Oxide
Klph: Klotholactase-phlorizin hydrolase
NHANES: National Health and Nutrition Examination Survey
NOS2: Nitric oxide synthase
NO: Nitric oxide
NCHS: Centers for Disease Control and Prevention
WBC: White blood cell
NLR: Neutrophil percentage/lymphocyte ratio
ALT: Alanine Aminotransferase
AST: Aspartate Aminotransferase
BUN: Blood urea nitrogen
FEV1: Forced expiratory volume in the first second
FVC: Forced vital capacity
PEF: Peak expiratory flow
FEF25%-75%: Forced expiratory flow at 25-75% of FVC
FEV1/FVC: The ratio of FEV1 to FVC
BMI: Body mass index
Ppb: Parts per billion
LDH: Lactate dehydrogenase
iNOS: Inducible NO synthase
TNF-α: Tumor necrosis factor alpha
CBC: Complete Blood Count
FGF23: Fibroblast growth factor 23
ASL: Airway surface liquid
OSA: Obstructive sleep apnoea
IGF-1: Insulin-like growth factor 1
